# The influence of growth mindset on perceived learning gain among Chinese college students: the mediating role of academic self-efficacy

**DOI:** 10.1186/s40359-026-04876-w

**Published:** 2026-06-11

**Authors:** Bingyou Ai, Yuqun Meng

**Affiliations:** 1 Education Research Institute, Xiamen University, Xiamen, Fujian 361005 China; 2 School of Marxism, Zhejiang College of Security Technology, Wenzhou, Zhejiang 325016 China; 3 School of International Law, Southwest University of Political Science and Law, Chongqing, 401120 China

**Keywords:** Growth mindset, Academic self-efficacy, Learning gain, Mediation effect, Chinese college students, Higher education

## Abstract

Growth mindset has attracted considerable scholarly attention as a predictor of academic outcomes, yet the psychological pathways linking it to students’ perceived learning gain remain insufficiently understood, particularly in Chinese higher education settings. This study examines the association between growth mindset and learning gain among Chinese college students and investigates whether academic self-efficacy serves as a mediating mechanism. A cross-sectional survey was administered to 478 undergraduates from three universities in Hubei Province, central China, using validated instruments for growth mindset, academic self-efficacy, and learning gain. Structural equation modeling with bias-corrected Bootstrap resampling was used to test the hypothesized mediation model. Results indicated that growth mindset was positively associated with learning gain, and that academic self-efficacy partially mediated this association. These findings integrate Dweck’s implicit theory of intelligence with Bandura’s self-efficacy framework within a single analytical model, offering evidence that growth mindset relates to learning gain both directly and indirectly through its association with academic self-efficacy. Practical implications for designing mindset and self-efficacy interventions in Chinese universities are discussed.

## Introduction

Over the past two decades, the concept of growth mindset—originally proposed by Carol Dweck—has attracted substantial scholarly attention across educational psychology and related disciplines [1]. At its core, growth mindset refers to the belief that intellectual abilities and talents are malleable qualities that can be cultivated through sustained effort, deliberate practice, and effective learning strategies, rather than fixed traits determined at birth [2]. This theoretical framework has reshaped how researchers and practitioners understand student motivation, resilience, and academic achievement, particularly within higher education contexts where learners face increasingly complex cognitive and affective demands.

Parallel to developments in mindset research, a growing body of work in China and internationally has turned to the construct of learning gain (or “learning sense of gain”) as a meaningful indicator of educational quality [3]. Unlike conventional outcome metrics such as grade point averages or graduation rates, learning gain captures the subjective perception of knowledge acquisition, skill development, and personal growth that students experience throughout their academic journey [4]. Chinese higher education reform, in particular, has elevated learning gain to a prominent position in quality assurance frameworks, viewing it as a student-centered criterion that reflects the depth and authenticity of the learning experience [5]. This shift responds to persistent criticism that traditional assessment approaches overlook the lived, affective dimensions of student development. Consequently, understanding which psychological factors shape learning gain has become an urgent empirical question for both researchers and policymakers.

Existing studies have begun to link growth mindset to a range of positive educational outcomes. Learners who endorse growth beliefs tend to adopt mastery-oriented goals, persist through academic setbacks, and engage more deeply with challenging material [6]. Several cross-sectional and longitudinal investigations have further demonstrated that growth mindset positively predicts academic performance and learning satisfaction among college students [7]. However, the bulk of this evidence originates from Western educational settings, and the psychological pathways through which growth mindset translates into perceived learning gain have not been adequately tested in other cultural contexts. The Chinese university context presents a particularly valuable site for such investigation—not because we assume collectivist values or examination-driven cultures necessarily moderate these effects, but because the absence of direct empirical evidence from Chinese higher education samples leaves open the question of whether the mediation structures identified in Western populations replicate in a different educational and cultural milieu. Testing well-established theoretical models in new populations is itself a meaningful scientific contribution, as it clarifies the boundary conditions and generalizability of those models [8, 9].

One candidate mechanism that warrants careful investigation is academic self-efficacy—the confidence students hold regarding their capacity to organize and execute the actions required for academic success [10]. Rooted in Bandura’s social cognitive theory, academic self-efficacy has been consistently identified as a robust predictor of student engagement, persistence, and achievement [11]. A number of researchers have reported that growth mindset fosters higher self-efficacy by encouraging students to interpret challenges as opportunities for skill refinement rather than evidence of inadequacy [12]. Meanwhile, academic self-efficacy itself has shown strong associations with learning satisfaction and perceived competence gains, suggesting that it may serve as a critical bridge between mindset orientations and the subjective experience of learning gain [3].

Despite these promising theoretical connections, the literature reveals notable gaps that motivate the present work. While the bivariate links among growth mindset, academic self-efficacy, and learning outcomes have been documented individually, the field lacks an integrated empirical test that simultaneously situates all three constructs within a single mediation framework [13]. Examining these variables jointly is theoretically important because it allows us to decompose the total association between mindset and learning gain into direct and indirect components, thereby specifying the psychological mechanism rather than merely confirming a correlational pattern. Furthermore, the mediating role of academic self-efficacy in this particular relationship has not been tested among Chinese university students, whose educational socialization, institutional structures, and assessment norms differ in ways that could plausibly alter the strength or pathway of these associations. Much of the existing research also relies on simple correlation or regression designs that do not capture indirect pathways. These gaps constrain both theoretical refinement and practical application, as educators and institutions currently lack evidence regarding whether and how mindset interventions might operate through self-efficacy to improve students’ perceived learning gains.

The present study seeks to address these shortcomings by constructing and testing a mediation model in which academic self-efficacy operates as an intermediary variable linking growth mindset to learning gain among Chinese college students. We pursue three interrelated objectives: first, to examine the direct effect of growth mindset on learning gain; second, to assess whether academic self-efficacy mediates this relationship; and third, to quantify the relative contributions of direct and indirect pathways. To achieve these aims, the study employs a questionnaire-based cross-sectional design, drawing on validated measurement instruments and applying structural equation modeling techniques to analyze mediation effects [14].

From a theoretical standpoint, this research contributes to the literature by integrating Dweck’s mindset theory with Bandura’s self-efficacy framework under a unified analytical model, thereby clarifying the cognitive-motivational chain through which belief systems shape experiential learning outcomes. From a practical perspective, the findings are expected to offer actionable insights for higher education institutions seeking to design targeted interventions—such as mindset training workshops or self-efficacy enhancement programs—that improve students’ authentic sense of learning gain. This dual significance underscores the timeliness and necessity of the present inquiry, especially as Chinese universities continue to prioritize student-centered quality evaluation.

In terms of contribution, this study offers three specific advances. It provides the first integrated empirical test of these three constructs within a single mediation model in a Chinese higher education sample. It moves beyond simple associational analysis by adopting a structured mediation approach that allows for decomposition of direct and indirect effects. And it extends existing scholarship by situating well-established psychological constructs within a Chinese university sample, thereby testing the cross-cultural applicability of the proposed mediation structure [7]. The remainder of this paper proceeds as follows: the second section reviews the theoretical foundations and develops research hypotheses; the third section describes the research design and methodology; the fourth section presents empirical results; and the final section discusses implications, limitations, and directions for future research.

## Theoretical foundations and hypotheses

### Core concept definitions and theoretical foundations

Growth mindset, as articulated within Dweck’s implicit theory of intelligence, denotes an individual’s belief that cognitive abilities are not innate endowments but rather capacities amenable to development through effort and appropriate strategy [15]. This stands in contrast to a fixed mindset, where intelligence is perceived as stable and largely immutable. Dweck’s framework distinguishes these two orientations along a continuum, arguing that where a person falls on this spectrum profoundly shapes how they interpret failure, approach challenges, and regulate learning behavior [2]. In empirical research, growth mindset is typically measured through self-report scales—most notably the Implicit Theories of Intelligence Scale—that assess the degree to which respondents endorse incremental versus entity views of ability [16].

Learning gain, the second construct central to this study, refers to students’ subjective appraisal of the knowledge, competencies, and personal development they have acquired during a defined period of education [17]. Unlike objective performance indicators, learning gain foregrounds the experiential and reflective dimension of academic growth. Its theoretical roots draw on both constructivist learning theory and the broader discourse on student-centered quality evaluation in higher education [5]. Scholars generally conceptualize learning gain as a multidimensional construct encompassing cognitive gains, affective development, and social skill advancement, each of which can be captured through structured survey instruments [18].

Academic self-efficacy, the third variable under examination, originates from Bandura’s social cognitive theory and refers specifically to learners’ confidence in their capacity to accomplish academic tasks and meet scholastic demands [11]. Bandura identified four principal sources feeding into self-efficacy beliefs: mastery experience, vicarious observation, verbal persuasion, and physiological-emotional states [19]. Within educational settings, academic self-efficacy has been operationalized across dimensions such as learning self-efficacy and behavioral self-efficacy, with measurement scales widely validated in both Western and Chinese student populations [20]. What makes this construct theoretically compelling for the present study is its dual positioning—it functions simultaneously as an outcome shaped by deeper belief systems like mindset orientation, and as a motivational driver that channels effort toward tangible learning outcomes. This intermediary character renders academic self-efficacy a natural candidate for mediating the relationship between growth mindset and learning gain, a possibility we formalize in the hypotheses that follow.

### Literature review on inter-variable relationships

Empirical evidence linking growth mindset to learning-related outcomes has accumulated steadily, though the specific connection to learning gain remains underexplored. Several studies have shown that students endorsing incremental beliefs about intelligence report greater academic satisfaction and deeper engagement with course material [21]. In Chinese educational samples, research has linked growth mindset to students’ perceived cognitive and affective development [22], although that particular study focused on primary school children and the generalizability of its findings to college-age populations warrants caution. Yet most of this work treats learning gain as a secondary outcome rather than a focal dependent variable, leaving the precise strength and mechanism of this association unclear.

The relationship between growth mindset and academic self-efficacy rests on firmer empirical ground. Longitudinal research has demonstrated that students who view ability as improvable gradually develop stronger confidence in their academic competence, partly because they reframe setbacks as informative rather than threatening [23]. Experimental mindset interventions have produced measurable increases in self-efficacy scores among undergraduate populations [24]. Cross-cultural studies further suggest this pattern holds across both individualist and collectivist educational contexts, although effect sizes vary depending on disciplinary domain and measurement instruments employed [25]. One point of contention concerns directionality—some scholars argue the relationship may be reciprocal, with high self-efficacy reinforcing incremental beliefs over time [26].

Turning to the self-efficacy–learning gain link, the evidence is largely convergent. Students with robust academic self-efficacy tend to set more ambitious learning goals, persist longer when confronting difficulty, and ultimately report higher levels of perceived growth [27]. A meta-analytic review confirmed that self-efficacy ranks among the strongest psychological predictors of subjective learning outcomes in postsecondary settings [28]. Research conducted within Chinese universities echoes these findings, revealing significant positive correlations between self-efficacy dimensions and multiple facets of learning gain [29].

What remains absent from the existing literature, however, is a single analytical model that simultaneously examines all three constructs and decomposes direct and indirect effects. While the bivariate links reviewed above are well documented, the question of whether academic self-efficacy accounts for a portion of the association between growth mindset and learning gain has not been directly tested in Chinese university populations [30]. Constructing and testing such an integrative model yields unique theoretical value by quantifying the relative magnitude of direct versus indirect pathways and by evaluating whether the mediation structure holds in a population where it has not previously been examined. This gap constitutes the theoretical entry point for the present study.

### Research hypotheses and theoretical model

Drawing on the theoretical foundations and empirical evidence reviewed above, we propose three interrelated hypotheses that collectively define the analytical framework of this study.

Given that growth mindset orients students toward effort-based interpretations of academic progress and encourages deeper engagement with learning tasks [6, 21], it is reasonable to expect a direct positive influence on perceived learning gain [22]. Accordingly, we advance:

#### H1

Growth mindset has a significant positive association with learning gain among college students.

The literature further indicates that incremental beliefs about intelligence cultivate stronger academic self-efficacy by reshaping how students appraise challenges and interpret performance feedback [23]. Students who hold growth-oriented views are more likely to build confidence in their academic capabilities over time. We therefore propose:

#### H2

Growth mindset has a significant positive association with academic self-efficacy among college students.

Finally, and most central to this investigation, the reviewed evidence suggests that academic self-efficacy may function as a psychological conduit through which mindset beliefs reach subjective learning outcomes. When growth mindset bolsters a student’s confidence, that heightened self-efficacy in turn promotes goal-directed behavior, sustained effort, and ultimately a richer sense of learning gain [11]. This reasoning leads to our mediation hypothesis:

#### H3

Academic self-efficacy mediates the association between growth mindset and learning gain—that is, growth mindset is indirectly associated with learning gain through its link with academic self-efficacy.

Together, these three hypotheses specify a partial mediation model in which growth mindset affects learning gain both directly (H1) and indirectly via academic self-efficacy (H2 and H3). The subsequent sections describe the research design and statistical procedures adopted to test this model empirically.

## Methods

### Participants and data collection

This study adopted a stratified convenience sampling approach to recruit undergraduate students from three universities in Hubei Province, central China, encompassing one comprehensive research university, one provincial-level institution, and one applied technology-oriented college [31]. The goal was to ensure reasonable representation across institutional types, academic years, and disciplinary backgrounds. Participants were required to be full-time undergraduates who had completed at least one semester of coursework, as a minimum exposure period was necessary for meaningful self-assessment of learning gain.

Questionnaires were distributed online via a professional survey platform during the spring semester of the 2024–2025 academic year. Class advisors from participating departments forwarded the survey link to student groups, and informed consent was obtained electronically before respondents could proceed [32]. A total of 520 questionnaires were collected over a three-week window. After removing responses with obvious patterns of inattention—such as identical answers across all items or completion times under 120 s—478 valid responses remained, yielding a recovery rate of 91.9% (valid responses out of total collected; the response rate relative to total invitations could not be precisely determined because the survey link was forwarded through class advisors, making the exact denominator unknown). This sample size comfortably exceeds the threshold recommended for structural equation modeling with three latent variables [33].

Table 1 summarizes the demographic profile of the final sample. The distribution reflects a reasonable gender balance and adequate coverage across grade levels and disciplinary categories.

**Table 1:** Demographic Characteristics of the Sample (*N* = 478)

Characteristic	Category	Frequency	Percentage (%)
Gender	Male	206	43.1
	Female	272	56.9
Grade	Freshman	98	20.5
	Sophomore	137	28.7
	Junior	132	27.6
	Senior	111	23.2
Discipline	Humanities and Social Sciences	189	39.5
	Science and Engineering	289	60.5

**Fig. 1:**
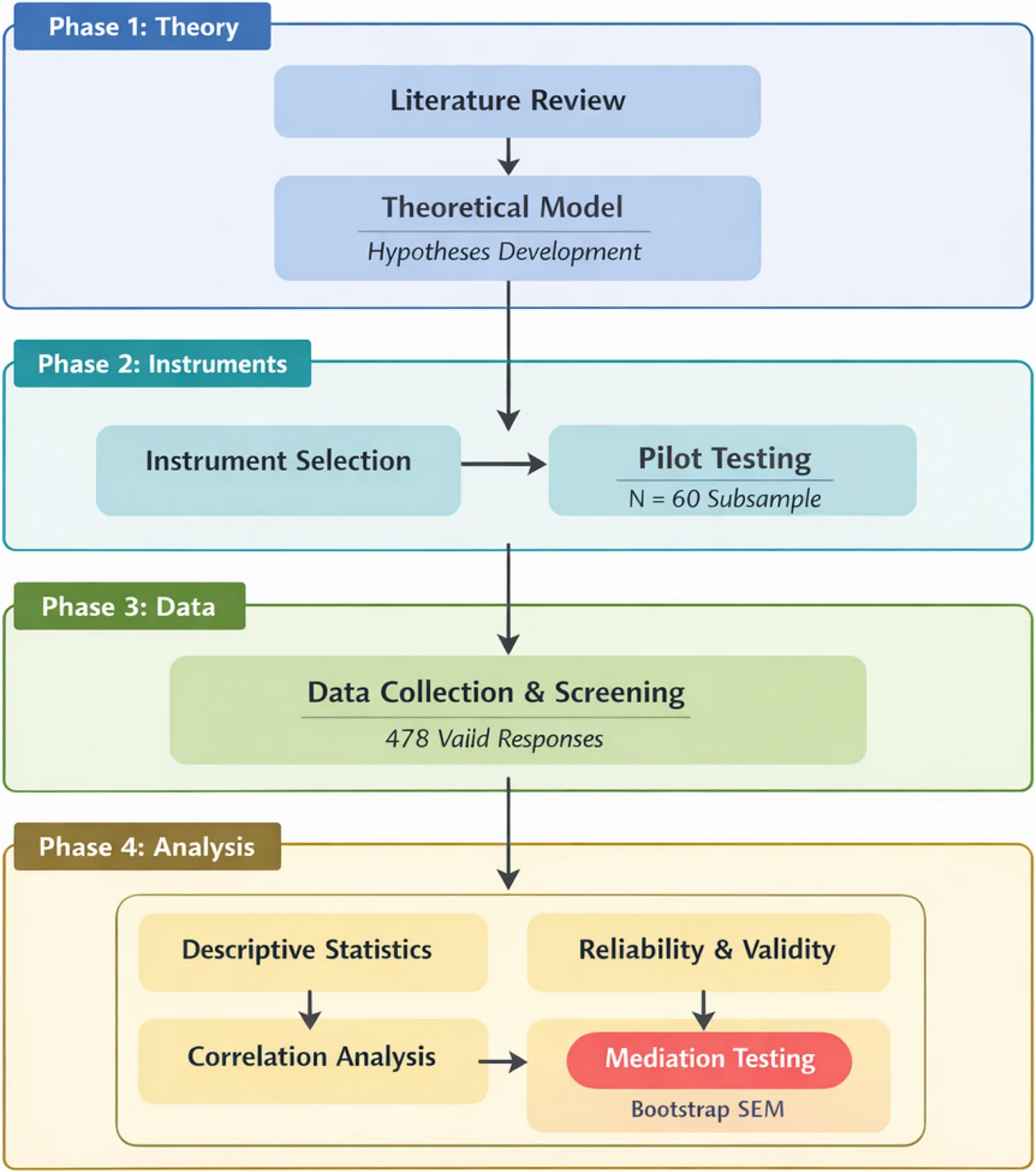
Overall Research Flowchart

As presented in Table 1, female students accounted for a slightly larger share of respondents, which is consistent with the general enrollment composition at the sampled institutions. Grade representation was relatively even, with sophomores and juniors comprising the largest portions. The overall research workflow—from instrument design through hypothesis testing—is depicted in Fig. 1.

Figure 1 illustrates the complete procedural sequence of this study, beginning with literature review and theoretical model construction, proceeding through instrument selection and pilot testing, followed by formal data collection and screening, and concluding with statistical analysis stages including descriptive statistics, reliability and validity assessment, correlation analysis, and mediation effect testing via structural equation modeling.

### Research instruments and variable measurement

Growth mindset was assessed using the 8-item Implicit Theories of Intelligence Scale developed by Dweck, with items rated on a 6-point Likert scale ranging from “strongly disagree” to “strongly agree” [16]. A validated Chinese adaptation of this instrument was adopted, which had undergone back-translation and cultural appropriateness review by a panel of three educational psychology experts [34]; the original English items and their adapted Chinese counterparts are set side by side in Appendix A (Table 6). Academic self-efficacy was measured with the Academic Self-Efficacy Questionnaire originally designed by Liang Yusong, comprising 22 items across two dimensions—learning ability self-efficacy and learning behavior self-efficacy—scored on a 5-point scale [35]; its full item set is given in Appendix B (Table 7). Learning gain was captured through a 20-item scale developed specifically for Chinese university contexts, covering three subdimensions: cognitive gain, affective gain, and social-communicative gain, also scored on a 5-point Likert format [36], with every item reproduced in Appendix C (Table 8). One point deserves emphasis here: each learning gain item asked respondents to weigh up their own development rather than to report a grade, so what we measure throughout is perceived, self-appraised gain and not documented attainment, and listing the items lets readers see exactly what students rated.

**Fig. 2:**
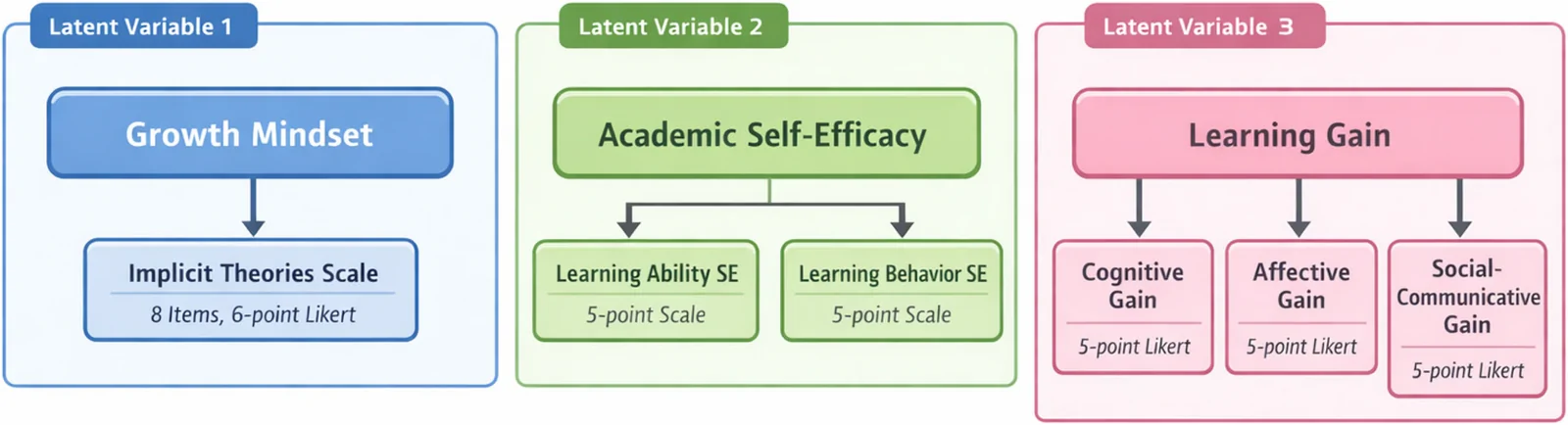
Variable Measurement Framework

The dimensional architecture of all three instruments and their correspondence to the theoretical constructs are depicted in Fig. 2.

Figure 2 illustrates how each latent variable maps onto its constituent dimensions and respective scale items, providing a visual overview of the measurement structure underlying the hypothesized model.

Prior to formal analysis, all instruments underwent pilot testing with a subsample of 60 students not included in the main study. Minor wording adjustments were made based on cognitive interview feedback to improve item clarity [37]. Confirmatory factor analysis was then conducted on the full sample to verify construct validity. Table 2 shows the reliability and validity indices obtained for each scale in this study.

**Table 2:** Reliability and Validity Test Results for Each Scale

Scale	Number of Items	Cronbach’s $\:\alpha\:$	CR	AVE
Growth Mindset Scale	8	0.903	0.911	0.563
Academic Self-Efficacy Scale	22	0.926	0.932	0.518
Learning Gain Scale	20	0.941	0.945	0.537

As presented in Table 2, all Cronbach’s $\:\alpha\:$ coefficients exceeded 0.90, indicating strong internal consistency. Composite reliability values surpassed the 0.70 benchmark, and average variance extracted for each construct exceeded the 0.50 threshold recommended by Fornell and Larcker, confirming adequate convergent validity [38]. Discriminant validity was also established, as the square root of each construct’s AVE exceeded its correlations with the other two latent variables [39]. These results collectively confirm that the measurement instruments performed well in the present sample and are suitable for subsequent structural modeling.

### Data analysis strategy

Data analysis proceeded through a sequential multi-step protocol. SPSS 26.0 handled descriptive statistics, common method bias testing, and correlation analysis, while AMOS 24.0 was employed for confirmatory factor analysis and structural equation modeling. First, Harman’s single-factor test was conducted to evaluate common method variance, given that all variables were collected via self-report in a single survey wave [40]. Next, confirmatory factor analysis assessed the measurement model fit, followed by Pearson correlation analysis to examine bivariate associations among the three core variables.

The mediation analysis adopted the approach outlined by Baron and Kenny, supplemented by the bias-corrected percentile Bootstrap method with 5,000 resamples to construct 95% confidence intervals for indirect effects [41]. The mediation model can be formally expressed through the following standard regression equations [42]:

1 $$\:M=a\times\:X+{e}_{1}$$

2 $$\:Y={c}^{{\prime\:}}\times\:X+b\times\:M+{e}_{2}$$

3 $$\:Y=c\times\:X+{e}_{3}$$

In these equations, * X* represents growth mindset, * M* denotes academic self-efficacy (the mediator), and * Y* refers to learning gain. Coefficient * a* captures the effect of growth mindset on the mediator; * b* represents the mediator’s effect on learning gain after controlling for growth mindset; $\:{c}^{{\prime\:}}$ is the direct effect; and * c* is the total effect. The indirect (mediated) effect equals the product $\:a\times\:b$. Mediation is supported when the Bootstrap 95% confidence interval for $\:a\times\:b$ does not contain zero [43].

Both the confirmatory factor analysis and the structural mediation model were evaluated against the same set of conventional fit thresholds: $\:{\chi\:}^{2}/df<3$, RMSEA * <0.08*, CFI * >0.90*, and TLI * >0.90*. Following established guidelines, the mediation effect size was quantified as the ratio of indirect to total effect ($\:a\times\:b/c$) to quantify the proportion of the total effect transmitted through the mediating pathway. The hierarchical regression used to test the direct predictive association takes the standard form in which learning gain is regressed on demographic controls in Step 1, with growth mindset added in Step 2, thereby isolating the unique explanatory contribution of the focal predictor beyond demographic factors. This comprehensive analytical sequence ensures both the robustness of measurement and the rigor of mediation inference in the sections that follow.

## Results

### Descriptive statistics and correlation analysis

Before examining variable relationships, Harman’s single-factor test was performed to assess common method bias. An exploratory factor analysis with all measurement items entered simultaneously yielded a first unrotated factor accounting for 28.63% of total variance, well below the 40% critical threshold [40]. This result provides an initial indication that common method variance may not severely distort the data. However, because Harman’s test has been criticized for its insensitivity and reliance on a crude threshold [40, 44], we supplemented it with a common latent factor (CLF) approach. Specifically, a single unmeasured latent factor was added to the confirmatory factor analysis model, with paths to all observed indicators. The standardized path estimates in the structural model changed by no more than 0.03 when the CLF was included compared with when it was omitted, suggesting that common method variance does not substantively alter the pattern of associations reported below.

Table 3 presents the descriptive statistics and Pearson correlation coefficients for the three core variables and their subdimensions.

**Table 3:** Descriptive Statistics and Correlation Matrix for Key Variables

Variable	M	SD	1	2	3	4
1. Growth Mindset	4.21	0.78	1			
2. Learning Ability Self-Efficacy	3.52	0.71	0.417**	1		
3. Learning Behavior Self-Efficacy	3.48	0.69	0.392**	0.653**	1	
4. Academic Self-Efficacy (Overall)	3.50	0.65	0.431**	0.908**	0.912**	1
5. Learning Gain (Overall)	3.67	0.72	0.462**	0.519**	0.487**	0.538**

*M* Mean, *SD* Standard Deviation

** p<0.01

As shown in Table 3, respondents reported moderately favorable scores on all three constructs. Because the growth mindset scale uses a 6-point response format while academic self-efficacy and learning gain employ 5-point scales, direct cross-variable mean comparisons are not meaningful. Within each scale, scores fell above the respective midpoints: growth mindset (M = 4.21, SD = 0.78, on a 6-point scale), academic self-efficacy (M = 3.50, SD = 0.65, on a 5-point scale), and learning gain (M = 3.67, SD = 0.72, on a 5-point scale). The two subdimensions of self-efficacy—learning ability and learning behavior—exhibited very similar mean scores, indicating a balanced self-assessment pattern among respondents.

Turning to the correlations, all pairwise associations reached significance at the * p<0.01* level. Growth mindset showed moderate positive correlations with both overall academic self-efficacy (* r* = 0.431) and overall learning gain (* r* = 0.462). The strongest bivariate association emerged between academic self-efficacy and learning gain (* r* = 0.538), which aligns with prior findings that confidence in one’s academic capabilities closely tracks perceived educational outcomes [45]. These correlation patterns provide preliminary support for the hypothesized relationships and justify proceeding with mediation analysis, though correlation alone cannot establish directional or causal claims.

**Fig. 3:**
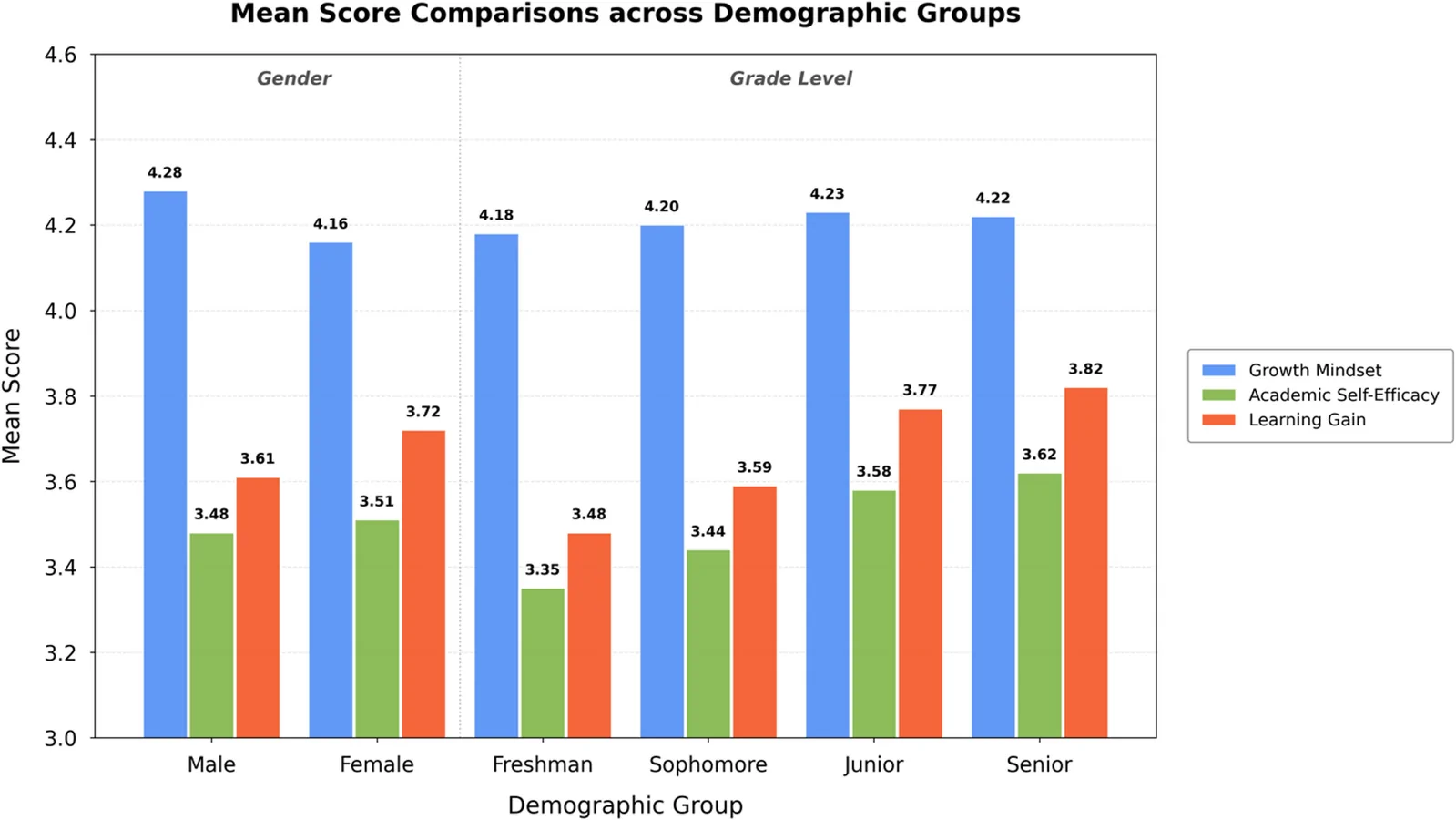
Mean Score Comparisons across Demographic Groups

To visualize group-level differences, mean scores on the three variables were compared across gender and grade level. Figure 3 depicts these comparisons.

Figure 3 demonstrates that female students scored slightly higher than males on learning gain, while male students reported marginally stronger growth mindset endorsement—though these gender differences were modest in magnitude. A more notable pattern appeared across grade levels: juniors and seniors reported higher learning gain and academic self-efficacy than freshmen and sophomores, a trend that likely reflects accumulated academic experience and progressive skill consolidation over time [46]. Growth mindset scores, by contrast, remained relatively stable across all four grades, suggesting that these beliefs may crystallize early and show less developmental fluctuation during the undergraduate years. These descriptive patterns set the stage for the structural modeling reported in the following sections.

### Regression analysis and direct effect testing

Hierarchical regression analysis was conducted following the model specification described in Sect.  3.3. Demographic variables—gender, grade, and discipline—were entered as controls in Step 1, and growth mindset was added in Step 2.

4 $$\:Y={\beta\:}_{0}+{\beta\:}_{1}{X}_{1}+{\beta\:}_{2}{X}_{2}+\cdots\:+{\beta\:}_{k}{X}_{k}+\epsilon\:$$

where * Y* denotes learning gain, $\:{X}_{1}$ through $\:{X}_{k}$ represent the predictor variables entered sequentially, $\:{\beta\:}_{k}$ are the corresponding regression coefficients, and $\:\epsilon\:$ is the error term.

Table 4 reports the results of the hierarchical regression.

**Table 4:** Hierarchical Regression Results for Learning Gain

Variable	Step 1 $\:\beta\:$	Step 1 * t*	Step 2 $\:\beta\:$	Step 2 * t*	VIF
Gender	0.067	1.48	0.054	1.27	1.03
Grade	0.113**	2.51	0.089*	2.10	1.05
Discipline	-0.041	-0.91	-0.023	-0.54	1.02
Growth Mindset	—	—	0.436***	10.52	1.04
$\:{R}^{2}$	0.021		0.221		
$\:\varDelta\:{R}^{2}$	0.021		0.200***		

* * p<0.05*; ** * p<0.01*; *** * p<0.001*. $$\:\beta\:$$ = standardized coefficient

The results in Table 4 indicate that demographic controls in Step 1 explained only 2.1% of the variance in learning gain, with grade emerging as the sole significant predictor ($\:\beta\:$ = 0.113, * p<0.01*). Once growth mindset entered the model in Step 2, the explained variance jumped to 22.1%—a substantial increment of 20.0% (* p<0.001*). The standardized regression coefficient for growth mindset was 0.436 (* t* = 10.52, * p<0.001*), confirming a robust positive direct effect on learning gain even after controlling for demographic characteristics. All variance inflation factor values remained well below 5, ruling out multicollinearity concerns [47]. These findings provide strong support for H1.

**Fig. 4:**
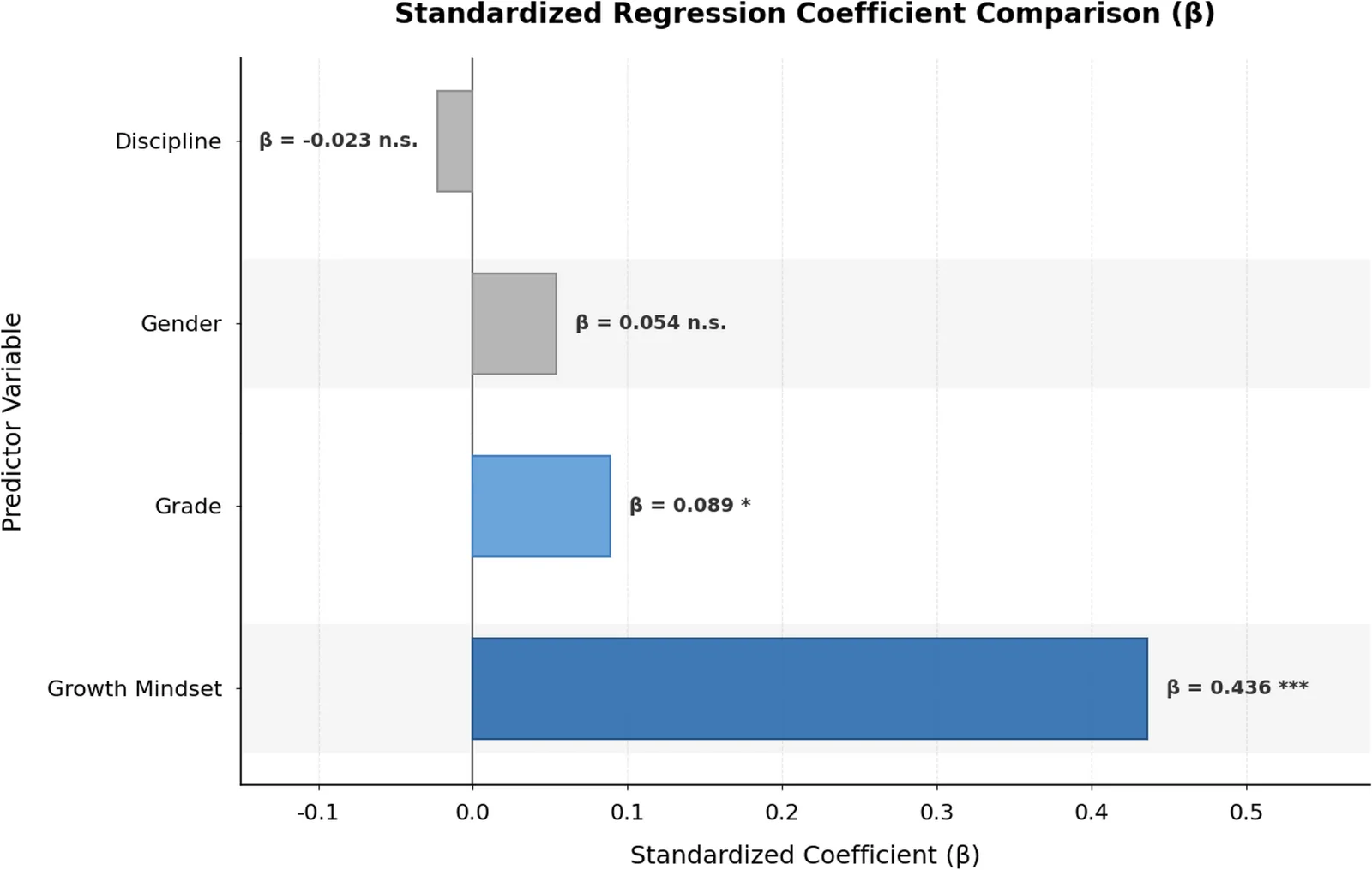
Standardized Regression Coefficient Comparison

To make the relative magnitudes of each predictor’s contribution visually accessible, Fig. 4 compares the standardized path coefficients from Step 2.

Figure 4 (produced using SPSS 26.0) presents the standardized $\:\beta\:$ coefficients for all predictors in the full model. Growth mindset stands out with a coefficient markedly larger than those of any demographic variable, underscoring its dominant role in predicting learning gain. Gender and discipline showed negligible, nonsignificant effects, while grade retained a modest but significant association—a pattern consistent with the descriptive trends observed in Fig. 3. The clear predominance of growth mindset as a predictor reinforces the theoretical rationale for examining the psychological mechanisms through which this belief orientation shapes perceived learning outcomes, an analysis we turn to next.

### Mediation effect testing of academic self-efficacy

Before presenting the mediation results, it is worth noting that the standardized coefficient for growth mindset predicting learning gain was 0.436 in the regression analysis above, which represents the total association between the two variables. In the structural equation model reported below, the direct path coefficient decreases to 0.248; this reduction occurs precisely because the mediator (academic self-efficacy) now absorbs a portion of that total association, which is the expected pattern when mediation is present. To formally test whether academic self-efficacy mediates the association between growth mindset and learning gain, structural equation modeling was conducted with bias-corrected Bootstrap resampling (5,000 iterations). The structural model demonstrated acceptable fit: $\:{\chi\:}^{2}/df$ = 2.37, RMSEA = 0.054, CFI = 0.952, TLI = 0.943—all within conventional thresholds. The proportion of the total effect attributable to the indirect pathway was calculated as:

5 $$\:\mathrm{M}\mathrm{e}\mathrm{d}\mathrm{i}\mathrm{a}\mathrm{t}\mathrm{i}\mathrm{o}\mathrm{n}\mathrm{R}\mathrm{a}\mathrm{t}\mathrm{i}\mathrm{o}=\frac{a\times\:b}{c}=\frac{a\times\:b}{a\times\:b+{c}^{{\prime\:}}}$$

Table 5 summarizes the decomposition of total, direct, and indirect effects along with their Bootstrap confidence intervals.

**Table 5:** Bootstrap Test Results for Mediation Effects

Effect Path	Point Estimate	SE	95% CI Lower	95% CI Upper
Total Effect (c): GM → LG	0.427	0.042	0.346	0.511
Direct Effect (c’): GM → LG	0.248	0.041	0.169	0.330
Indirect Effect (a×b): GM → ASE → LG	0.179	0.028	0.128	0.237
Path a: GM → ASE	0.389	0.039	0.314	0.468

*GM * Growth Mindset, *ASE *Academic Self-Efficacy, *LG * Learning Gain, *CI * Confidence Interval, *SE* Standard Error

5,000 Bootstrap samples

The results in Table 5 reveal several key findings. The total effect of growth mindset on learning gain was 0.427, and its 95% confidence interval excluded zero, confirming the overall predictive relationship. More critically, the indirect effect through academic self-efficacy reached 0.179, with a confidence interval of [0.128, 0.237]—clearly not containing zero—thereby establishing significant mediation [43]. The direct effect remained significant as well (point estimate = 0.248, 95% CI [0.169, 0.330]), which means academic self-efficacy does not fully absorb the influence of growth mindset on learning gain. The mediation is therefore partial rather than complete. Applying Eq. (5), the mediation ratio equals 0.179/0.427 = 41.9%, indicating that roughly two-fifths of the total effect operates through the self-efficacy pathway, while the remaining 58.1% reflects the direct channel from growth mindset to learning gain.

**Fig. 5:**
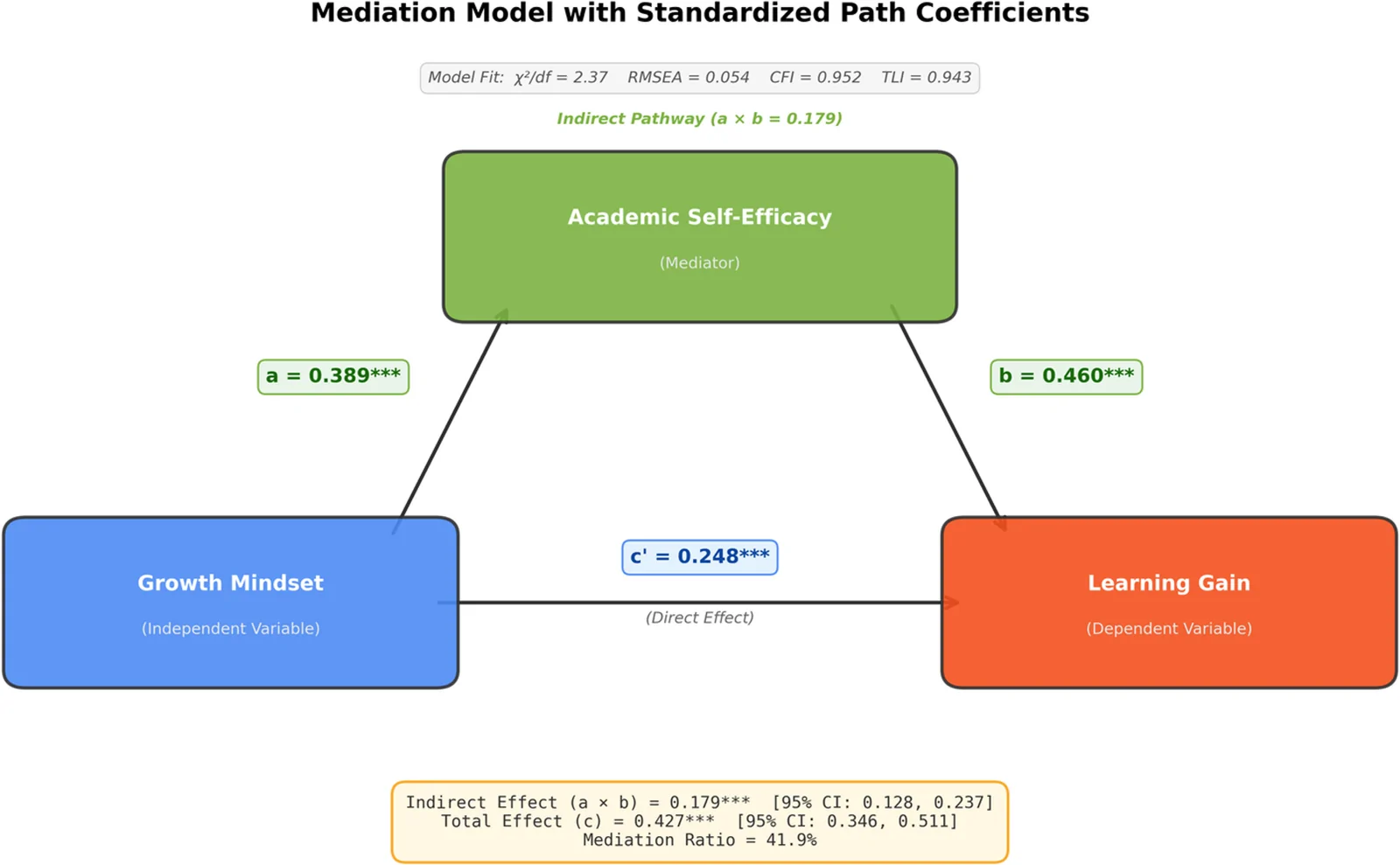
Mediation Model with Standardized Path Coefficients

The path coefficient for growth mindset predicting academic self-efficacy (path * a* = 0.389) and the coefficient for academic self-efficacy predicting learning gain after controlling for growth mindset (path * b* = 0.460, derived from $\:a\times\:b/a$) were both substantial and significant. Figure 5 presents the final structural model with all standardized path coefficients.

Figure 5 (produced using AMOS 24.0) illustrates the complete mediation structure, with growth mindset exerting a direct effect of 0.248 on learning gain and an indirect effect of 0.179 routed through academic self-efficacy. The model makes clear that while growth mindset retains a meaningful direct link to perceived learning outcomes, a considerable portion of its influence is channeled through the confidence students develop in their academic capabilities. To evaluate the robustness of the hypothesized model, two alternative models were tested. First, a reverse mediation model in which academic self-efficacy served as the predictor and growth mindset as the mediator yielded poorer fit and a non-significant indirect pathway. Second, a parallel mediator model with the two subdimensions of self-efficacy (learning ability and learning behavior) entered as separate mediators showed acceptable fit but did not improve upon the hypothesized model in terms of parsimony or explanatory power; both subdimensions carried significant indirect effects, supporting the decision to model self-efficacy as a unitary construct. These comparisons strengthen confidence in the hypothesized directional structure. Taken together, these results collectively support H2 and H3: growth mindset significantly predicts academic self-efficacy, and academic self-efficacy partially mediates the growth mindset–learning gain relationship. The theoretical and practical implications of this mediation pattern are discussed in the following section.

## Discussion

The empirical findings of this study converge on a clear narrative: growth mindset is positively associated with college students’ learning gain, and academic self-efficacy partially accounts for this association. What follows is an attempt to unpack the psychological logic behind these results, situate them within the broader research landscape, and draw out their practical implications for higher education.

The significant direct effect of growth mindset on learning gain ($\:\beta\:$ = 0.248 in the structural model) is not, on its own, surprising—but the magnitude warrants reflection. Why should a belief about the malleability of intelligence translate so readily into a richer subjective sense of having learned? One plausible explanation centers on how growth-oriented students engage with the learning process itself. When a student views intellectual capacity as expandable, academic tasks cease to function purely as evaluations of fixed competence and instead become opportunities for genuine development. This reframing, consistent with Dweck and Yeager’s (2019) account of how mindset shapes behavioral engagement [6], alters the quality of attention, the willingness to grapple with confusion, and the depth of processing applied to new material. A student who believes effort matters is more likely to persist through a difficult problem set, seek clarification from instructors, or revisit lecture content—and these behaviors accumulate over weeks and months into a tangible sense of cognitive and personal progress. Put differently, growth mindset does not simply correlate with learning gain; it appears intertwined with the very engagement patterns through which gain is generated and perceived.

Perhaps more revealing is the finding that 41.9% of the total effect passes through academic self-efficacy. This proportion suggests that mindset beliefs do not appear to relate to learning gain exclusively through direct channels but seem substantially routed through the confidence students build in their own academic capabilities. The mechanism here seems intuitive once articulated: students who endorse incremental views of intelligence gradually develop a stronger sense that they can handle academic demands—not because they ignore difficulty, but because they interpret difficulty as a signal that growth is occurring rather than as proof of inadequacy. This shift in self-appraisal feeds into what Bandura [19] would recognize as mastery experience reinterpretation. Each small academic success, and even each constructive failure, reinforces the conviction that one’s efforts are productive. That accumulating self-efficacy then accompanies further engagement, goal-setting, and persistence, all of which amplify the student’s perception that meaningful learning has taken place.

The partial—rather than complete—nature of this mediation deserves attention as well. That growth mindset retains a direct path to learning gain even after accounting for self-efficacy indicates additional mechanisms at work. We suspect that mindset beliefs also influence learning gain through affective and motivational pathways not captured by self-efficacy alone. For instance, growth-oriented students may experience greater intrinsic enjoyment of intellectual challenge, develop stronger academic identity, or cultivate more adaptive emotional regulation strategies during stressful academic periods. These channels, while beyond the scope of the current model, represent promising avenues for future decomposition of the mindset–outcome relationship.

How do these results compare with existing scholarship? The direct relationship between growth mindset and positive academic outcomes aligns well with the broader literature, including studies conducted in both Western and East Asian educational contexts [7, 25]. Where our findings contribute is in specifying learning gain—rather than GPA or course grades—as the outcome, and in demonstrating that the mindset–gain link operates partially through self-efficacy in a Chinese university sample. Some prior work has found stronger total effects of mindset on achievement-related outcomes, which may reflect differences in how objective performance versus subjective gain respond to belief systems. Objective grades are shaped by numerous external factors [28]—exam design, grading norms, peer competition—that dilute the influence of any single psychological variable. Subjective learning gain, by contrast, is more directly tethered to the student’s internal experience, which is precisely where mindset beliefs exert their most immediate influence. This distinction may explain why the effect sizes observed here, while moderate, reveal a clean and theoretically coherent pattern.

One point of potential divergence with prior research concerns the stability of growth mindset across grade levels, as observed in the descriptive analysis. While some studies have reported that mindset beliefs shift during the college years [23]—sometimes becoming more fixed under competitive academic pressure—our data showed relatively flat growth mindset scores from freshman to senior year. This could reflect cultural factors specific to the Chinese educational context. Within Confucian educational traditions, the belief that diligence and sustained effort drive intellectual development has deep historical roots [8, 9]. Li (2012) argued that East Asian learning models prioritize self-perfection through persistent effort rather than the cultivation of innate ability—a cultural orientation that resonates with the incremental beliefs captured by Western growth mindset scales. Heine et al. (2001) similarly demonstrated that Japanese participants responded to failure with increased persistence, suggesting that effort-based beliefs may be more deeply embedded in East Asian populations. If Chinese students already hold relatively strong incremental beliefs as a baseline, this would explain both the high mean scores and the limited grade-level variation observed in our sample. We note, however, that the growth mindset scale used here was developed within a Western theoretical tradition, and it remains an open question whether this instrument captures the full nuance of effort-related beliefs as they operate within Chinese educational socialization. Future research should explore culturally grounded measures that distinguish between Western conceptions of growth mindset and Confucian-rooted effort beliefs. Alternatively, it may simply indicate that the cross-sectional design lacks the temporal resolution to detect subtle within-person changes that longitudinal tracking would reveal.

Turning to practical implications, these findings carry concrete relevance for university educators and administrators. If growth mindset predicts learning gain both directly and through self-efficacy, then interventions targeting mindset beliefs could yield compounding benefits. Brief, structured workshops that teach students about neuroplasticity [24] and reframe academic struggle as a normal part of intellectual development have shown promise in other settings and could be adapted for Chinese university classrooms. It is worth stressing how light such programming can be: the national experiment we drew on earlier delivered its mindset message in under an hour, entirely online and self-administered, yet still moved achievement among American high-school students [24]. A format that lean and inexpensive should travel well to Chinese universities, where the sheer size of student cohorts makes scalability a genuine constraint, and it could be piloted at orientation before any heavier intervention is built. Importantly, our results suggest that such interventions should not stop at mindset alone. Deliberately building academic self-efficacy—through scaffolded task design, formative feedback that highlights specific areas of improvement, and mentoring programs that provide vicarious success models [11, 19]—would amplify the downstream effects on learning gain.

Course design itself may also play a role. When instructors create assessment structures that reward process and improvement rather than solely final performance, they implicitly reinforce growth-oriented beliefs and give students concrete evidence of their own capability development. This kind of pedagogical alignment between mindset theory and classroom practice could be especially powerful in large lecture courses where students often feel anonymous and disconnected from their own progress.

Finally, institutional quality assurance frameworks that incorporate learning gain as a key metric would benefit from recognizing the psychological infrastructure that supports it. Growth mindset and academic self-efficacy are not luxuries or secondary concerns—they are foundational conditions that shape whether students actually feel they have learned something meaningful during their university years. Neglecting these internal processes while focusing exclusively on external performance indicators risks painting an incomplete picture of educational quality. More concretely, we recommend three actionable strategies grounded in the present findings. First, universities could embed brief growth mindset modules into freshman orientation programs, drawing on evidence from large-scale mindset interventions [24] and adapting them to Chinese institutional norms. Second, course-level assessment redesign—for instance, replacing single high-stakes examinations with cumulative portfolio systems and structured formative feedback loops—would provide students with recurring evidence of their own competence development, thereby reinforcing both self-efficacy and perceived learning gain. Third, faculty development initiatives should equip instructors with strategies for delivering effort-focused praise and process-oriented feedback, both of which have been shown to strengthen self-efficacy beliefs in educational settings [11]. These interventions target the specific psychological pathways identified in the present mediation model and can be piloted and refined within existing institutional structures.

In sum, the discussion returns to a central insight: what students believe about the nature of their own abilities appears closely connected to what they ultimately feel they have gained from higher education. Academic self-efficacy serves as a critical amplifier in this process, translating abstract beliefs into the lived confidence that sustains effort, deepens engagement, and generates a genuine sense of intellectual growth.

## Conclusion

This study set out to examine how growth mindset influences learning gain among Chinese college students and whether academic self-efficacy serves as a mediating mechanism in that relationship. Drawing on survey data from 478 undergraduates across three universities, the analysis yielded three core findings. First, growth mindset is significantly and positively associated with learning gain, accounting for a substantial proportion of variance even after demographic factors are controlled. Second, growth mindset positively predicts academic self-efficacy, consistent with the view that incremental beliefs about intelligence are associated with greater confidence in one’s scholarly capabilities. Third, academic self-efficacy partially mediates the growth mindset–learning gain link, with approximately 41.9% of the total effect transmitted through this indirect pathway. All three hypotheses received empirical support.

From a theoretical standpoint, the contribution of this work lies in providing an integrated empirical test that connects two influential frameworks—Dweck’s implicit theory of intelligence and Bandura’s self-efficacy theory—within a single mediation model directed at a distinctly student-centered outcome. Most prior research has examined these constructs in isolation or paired them with conventional achievement indicators like grades. By foregrounding learning gain as the dependent variable, this study shifts attention toward the experiential quality of education rather than its quantifiable products. The identification of partial mediation is itself informative: it tells us that self-efficacy is an important but not exclusive conduit, signaling that additional psychological pathways remain to be mapped.

For practitioners, the implications are reasonably direct. Universities seeking to enhance students’ perceived learning outcomes would do well to invest in interventions that target both mindset beliefs and self-efficacy simultaneously. Growth mindset workshops, when paired with pedagogical designs that provide cumulative evidence of competence development—such as portfolio assessments, formative feedback loops, and structured reflection exercises—stand to produce compounding effects. The data also suggest that institutional attention to learning gain as a quality metric should be accompanied by concern for the psychological conditions that make such gain possible in the first place.

Several limitations temper the conclusions drawn here and should be acknowledged candidly. The cross-sectional design precludes causal inference. Although the mediation model specifies a directional pathway, the actual temporal ordering of changes in mindset, self-efficacy, and learning gain cannot be established from single-wave data. As Maxwell and Cole (2007) and Fiedler, Schott, and Meiser (2011) have demonstrated, cross-sectional mediation analyses can yield substantially biased estimates of longitudinal parameters [48, 49]. Readers should therefore interpret the reported indirect effects as conditional associations consistent with the hypothesized model rather than as evidence of a causal process. The sample, though adequate in size, was drawn from three universities in a single geographic region, which constrains generalizability to other institutional types and cultural contexts. Additionally, all variables were assessed through self-report questionnaires, and despite both Harman’s test and the supplementary common latent factor analysis suggesting no severe contamination [40, 44], shared method variance remains an inherent concern given that all three constructs were measured by the same respondents at the same time point. A related caveat is worth spelling out: because learning gain was captured purely as students’ own perception, we cannot say whether their objective attainment actually shifted. Future work would gain a great deal from setting self-reported gain alongside a hard achievement record—semester GPA being the obvious candidate—especially when an intervention is in play, since only that pairing can reveal whether a mindset shift moves real grades rather than merely how students feel about their progress [24, 28]. The model also considered only one mediator, yet the partial mediation finding itself hints that other variables—academic engagement, emotional regulation, goal orientation—likely play complementary roles that this study did not capture.

We also acknowledge that the direction of effects between growth mindset and academic self-efficacy may be reciprocal rather than unidirectional. Talsma et al. (2018) presented meta-analytic evidence for reciprocal causation between self-efficacy and academic performance [26], and a similar dynamic could operate between mindset and self-efficacy. Our cross-sectional design cannot adjudicate among these possibilities. Future research should address these gaps through several avenues. Longitudinal panel designs tracking students across multiple semesters would allow researchers to observe how mindset beliefs, self-efficacy, and learning gain co-evolve over time and to test for reciprocal causation. Cross-cultural comparisons between Chinese and Western student populations could clarify whether the mediation structure identified here is culturally universal or shaped by specific educational norms and socialization patterns. Finally, multiple mediator models that incorporate constructs such as learning engagement, academic resilience, or achievement goal orientation alongside self-efficacy would provide a more comprehensive picture of the psychological architecture connecting mindset beliefs to the lived experience of learning. Such extensions would not only refine the theoretical model proposed here but also yield richer guidance for educational practice.

## Data Availability

All data generated and analyzed during the current study are available from the corresponding author upon reasonable request.

## Appendix A. items of the implicit theories of intelligence scale

**Table 6:** Original and Adapted Items of the 8-Item Implicit Theories of Intelligence Scale

No.	Original Item (Dweck, 1999)	Adapted Chinese Version, back-translated (Wang & Zhang, 2020)
1	You have a certain amount of intelligence, and you really cannot do much to change it.	A person’s level of intelligence is largely fixed, and effort cannot change it by much.
2	Your intelligence is something about you that you cannot change very much.	My own level of intelligence is something I can hardly alter.
3	You can learn new things, but you cannot really change your basic intelligence.	I can acquire new knowledge, yet my underlying intelligence stays the same.
4	To be honest, you cannot really change how intelligent you are.	Honestly, how clever I am is not something I am able to change.
5	No matter who you are, you can significantly change your intelligence level.	Whoever they are, anyone can raise their intelligence a great deal through effort.
6	You can always substantially change how intelligent you are.	I can always improve my intelligence substantially as long as I work at it.
7	No matter how much intelligence you have, you can change it quite a bit.	However much intelligence a person starts with, it can still grow considerably.
8	You can change even your basic intelligence level considerably.	Even the most basic level of my intelligence can be developed markedly.

Note. Items 1–4 reflect a fixed (entity) view and are reverse-scored; items 5–8 reflect a growth (incremental) view. All items were rated on a 6-point Likert scale. Adapted items are English back-translations of the validated Chinese version [34]

## Appendix B. items of the academic self-efficacy questionnaire

**Table 7:** Items of the 22-Item Academic Self-Efficacy Questionnaire by Dimension

Dimension	No.	Item
Learning ability self-efficacy	1	I am confident that I can understand the most difficult material presented in my courses.
Learning ability self-efficacy	2	I believe I can master the knowledge and skills taught in my major.
Learning ability self-efficacy	3	I am able to work out the answers to challenging academic problems.
Learning ability self-efficacy	4	Compared with my classmates, I expect to perform well in my studies.
Learning ability self-efficacy	5	I am confident I can do an excellent job on assignments and examinations.
Learning ability self-efficacy	6	Even when course content is complex, I trust my ability to learn it.
Learning ability self-efficacy	7	I can grasp the core concepts of a subject fairly quickly.
Learning ability self-efficacy	8	I am sure I can meet the academic standards my teachers set.
Learning ability self-efficacy	9	When I put in the effort, I am capable of achieving good grades.
Learning ability self-efficacy	10	I have the intellectual ability needed to complete my degree successfully.
Learning ability self-efficacy	11	I am confident in my capacity to learn on my own when this is required.
Learning behavior self-efficacy	12	I can stick to a study plan until I finish it.
Learning behavior self-efficacy	13	I am able to keep concentrating during long study sessions.
Learning behavior self-efficacy	14	I can resist distractions such as my phone while studying.
Learning behavior self-efficacy	15	I manage my study time effectively across competing tasks.
Learning behavior self-efficacy	16	When I run into difficulties, I keep working rather than giving up.
Learning behavior self-efficacy	17	I can organize my notes and learning materials in a systematic way.
Learning behavior self-efficacy	18	I am able to set clear learning goals for myself each week.
Learning behavior self-efficacy	19	I can adjust my study methods when an approach is not working.
Learning behavior self-efficacy	20	I review material regularly instead of leaving it to the last minute.
Learning behavior self-efficacy	21	I can motivate myself to study even when a subject does not interest me.
Learning behavior self-efficacy	22	I am able to seek help from teachers or peers when I need it.

Note. All items were rated on a 5-point Likert scale. The instrument was originally developed by Liang Yusong [35]

## Appendix C. items of the learning gain scale

**Table 8:** Items of the 20-Item Learning Gain Scale by Subdimension

Dimension	No.	Item
Cognitive gain	1	My professional knowledge has expanded substantially during my studies.
Cognitive gain	2	I have improved my ability to analyze and solve problems.
Cognitive gain	3	I can now think more critically about academic questions.
Cognitive gain	4	My capacity for independent and creative thinking has grown.
Cognitive gain	5	I have become better at integrating knowledge across different subjects.
Cognitive gain	6	My ability to apply what I learn to real situations has increased.
Cognitive gain	7	I have developed stronger logical reasoning skills.
Cognitive gain	8	I have acquired research and inquiry skills I did not have before.
Affective gain	9	My interest in my field of study has deepened.
Affective gain	10	I feel a stronger sense of responsibility toward my own learning.
Affective gain	11	My confidence in facing academic challenges has grown.
Affective gain	12	I have developed clearer personal values and a sense of purpose.
Affective gain	13	My attitude toward lifelong learning has become more positive.
Affective gain	14	I feel greater satisfaction with my personal growth at university.
Social-communicative gain	15	I have become better at expressing my ideas clearly to others.
Social-communicative gain	16	My ability to work effectively in a team has improved.
Social-communicative gain	17	I am more comfortable communicating with teachers and classmates.
Social-communicative gain	18	I have strengthened my ability to cooperate toward shared goals.
Social-communicative gain	19	My skills in listening to and understanding others have grown.
Social-communicative gain	20	I am better able to resolve disagreements in a constructive way.

Note. All items were rated on a 5-point Likert scale and asked respondents to appraise their own perceived development. The instrument was developed for Chinese university contexts [36]

## Abbreviations

GM: Growth Mindset
ASE: Academic Self-Efficacy
LG: Learning Gain
SEM: Structural Equation Modeling
CR: Composite Reliability
AVE: Average Variance Extracted
CFI: Comparative Fit Index
TLI: Tucker-Lewis Index
RMSEA: Root Mean Square Error of Approximation
VIF: Variance Inflation Factor
CI: Confidence Interval
SE: Standard Error
SD: Standard Deviation
